# Primary Vaginal Adenocarcinoma of Intestinal-Type: A Case Report of a Rare Tumor With Review of Histology, Differential Diagnosis, and Literature

**DOI:** 10.7759/cureus.25298

**Published:** 2022-05-24

**Authors:** Ahmed Sabri, Changzhao Li, FNU Monika, Aproova Sharma, Poonam Sharma

**Affiliations:** 1 Pathology and Laboratory Medicine, Creighton University School of Medicine, Omaha, USA

**Keywords:** vaginal cancer, primary adenocarcinoma, adenocarcinoma, female, diethylstilbestrol, vagina, intestinal-type, vaginal adenocarcinoma

## Abstract

Intestinal-type adenocarcinoma is a rare primary vaginal carcinoma. Vaginal adenocarcinomas are most frequently a metastatic lesion, and less commonly, have clear cell histology and occur in young women with diethylstilbestrol (DES) exposure in utero. Due to the limited diagnostic power of immunohistochemistry (IHC) in differentiating primary from metastatic adenocarcinoma of the vagina, clinical and radiological correlation is critical in this scenario. The prognosis of this tumor depends on the patient’s age, tumor stage, tumor differentiation, lymph node status, and distant metastasis. Several treatment modalities are present depending on the tumor stage.

We present a case of primary adenocarcinoma of the vagina and describe the histopathologic features including the immunoprofile of the tumor and discuss the clinicopathologic features, differential diagnosis, diagnostic challenges, and a brief overview of the literature about age, size, site, immunohistochemical staining, and DES exposure.

## Introduction

Primary vaginal adenocarcinoma is a rare entity, comprising only 1% to 2% of all gynecologic malignancies [1]. The most common primary vaginal tumors are squamous cell carcinomas (80%), and adenocarcinoma (15%) [2]. Vaginal adenocarcinoma is most frequently a metastatic lesion, and less commonly, has clear cell histology and occurs in young women with diethylstilbestrol (DES) exposure in utero [3]. Primary vaginal clear cell adenocarcinoma is reported to have a genetic profile like that of ovarian clear cell adenocarcinoma [4]. Primary mucinous vaginal adenocarcinoma is rare but has been described in DES-exposed women [3]. Chromosomal and genetic characteristics of primary vaginal adenocarcinoma have not been well elucidated given the rarity of this case. The optimal treatment strategy for this disease is also unclear [4]. 

## Case presentation

**Figure 1:**
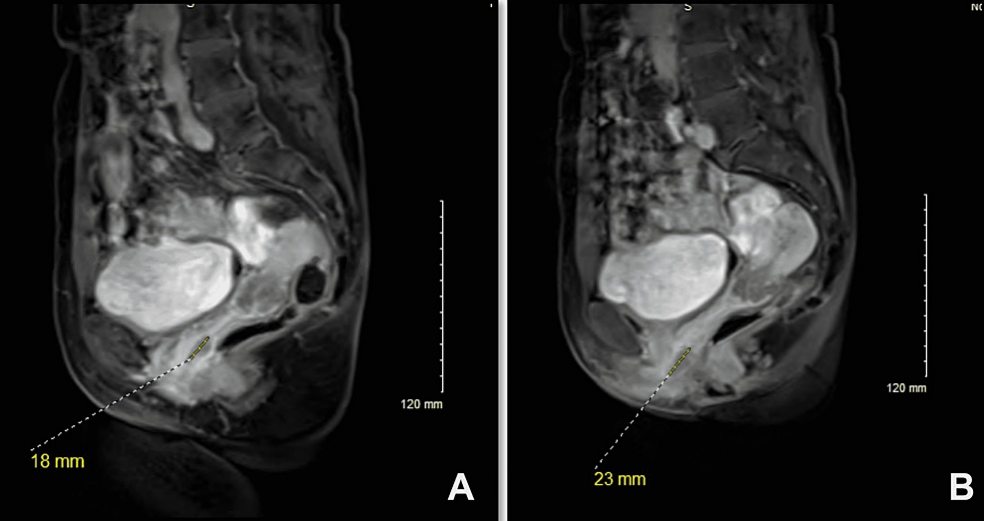
MRI of the pelvis A: The tumor is bilobed with a component of this tumor within the middle third of the vagina measuring 18 mm, B: There is a 2.3 cm mass involving the lower one-third of the vagina causing mass effect on and likely invading the distal female urethra

A 62-year-old postmenopausal para^0+2^ female presented at the outpatient department of a tertiary care hospital in Omaha with a two-month history of dysuria, postmenopausal bleeding (PMB), and associated cramping. She also had a history of vaginal atrophy for which she used an estrogen cream for a few days. General physical examinations including vital signs and abdominal exams were unremarkable. There was no abdominal tenderness or mass. A gynecologic exam revealed normal genitalia and anal region. On exam of the introitus, there was a fleshy friable but firm mass around the urethral meatus. On speculum exam, there was a 3 cm sessile, firm mass in the right upper quadrant of the vagina just inside the introitus. The cervix showed no lesion. On a bimanual exam, the uterus was slightly enlarged. An attempt was made to pass an endometrial biopsy catheter, however, it could not be passed easily. Therefore, an endometrial biopsy was not obtained. Before proceeding to obtain a sample of the endometrium for tissue diagnosis it was decided to consult a urology service for evaluation because the vaginal mass around was close to the urethral meatus. In the meantime, a transvaginal ultrasound was performed to reveal a symmetrical uterus with a uniform endometrial thickness of 0.50 cm. No other abnormality was noted. An MRI of the pelvis showed uterine intramural and subserosal fibroids, the largest measuring 3 cm. There was a 2.3 cm mass involving the lower one-third of the vagina causing a mass effect on the distal urethra. The tumor was bilobed with another component of this tumor within the middle third of the vagina measuring 1.8 cm (Figure 1, A) and a 2.3 cm mass involving the lower one-third of the vagina (Figure 1, B). No rectal invasion or pelvic lymphadenopathy was noted and the patient doesn't have any history of previous malignancies. No suspicious osseous lesions were identified. Findings were suspicious for stage IVa vaginal cancer and tissue sampling was recommended by radiology.

Examination under anesthesia followed by cystourethroscopy was performed by the urology team. On exam, the urethral opening was found to be nearly obliterated by a fungating mass at the anterior portion of the vagina. Using a flexible cystoscope, a urethral orifice located at about 11 o'clock in relation to the vaginal mass was identified. The urethra and external sphincter were uninvolved with the mass, but there was a significant mass effect pushing the urethra anterolaterally. The cystoscopy demonstrated a normal-appearing bladder without any concerning findings. A vaginal exam was performed to reveal two fungating masses, one near the urethra in the anterior vagina and one in the posterior vagina. The cervix showed no abnormality.

**Figure 2:**
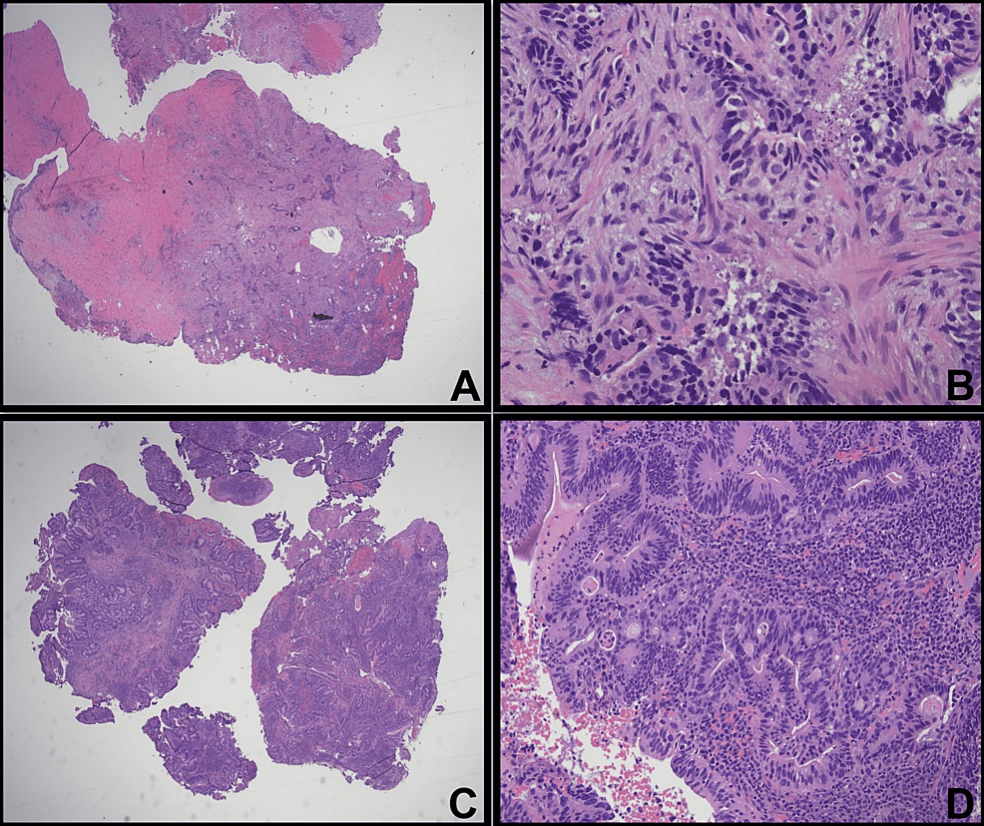
Hematoxylin and eosin (H&E) stains of the sections from the biopsy A,B: Posterior vaginal mass; C,D: Anterior vaginal periurethral mass

**Figure 3:**
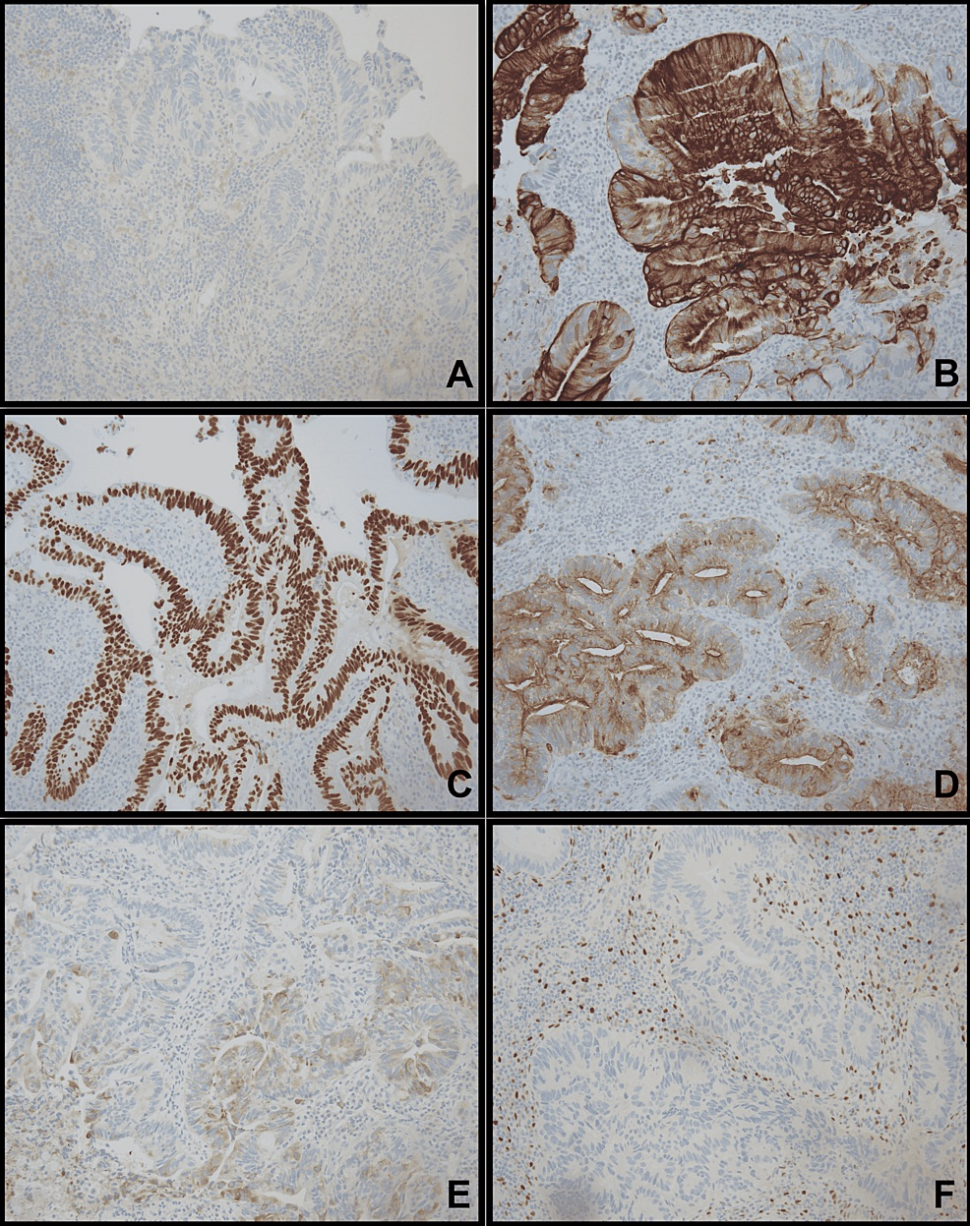
Immunohistochemical (IHC) stains Pictures showing the immunohistochemical staining of CK7 (A), CK20 (B), CDX2 (C), CEA (D), EMA (E), and ER (F) in sections of the anterior vaginal periurethral mass. Magnification, 200X. CK7: Cytokeratin 7, CK20: Cytokeratin 20, CDX2: Caudal-type homeobox 2, CEA: Carcinoembryonic antigen, EMA: Epithelial membrane antigen, ER: Estrogen receptor

A liquid-based pap smear was taken and both masses were separately biopsied for pathological examination. The patient's pap smear was negative for intraepithelial lesion or malignancy. The biopsies from posterior and anterior vaginal periurethral masses showed similar features on the histological examination. The tissue revealed moderately formed glands with a complex structure including a cribriform growth pattern that invaded the stroma. The glands were lined by atypical pseudostratified cuboidal to columnar cells with pleomorphic, hyperchromatic nuclei. There were scattered cells with intracytoplasmic mucin, but abundant extracellular mucin was not present. The tumor cells were mitotically active with frequent intraluminal necrosis. The overall morphology of these tumor cells resembled those of moderately differentiated intestinal adenocarcinoma (Figure 2, A-D). The tumor cells from both biopsy sites were positive for cytokeratin 20 (CK20), caudal-type homeobox 2 (CDX2), carcinoembryonic antigen (CEA), and epithelial membrane antigen (EMA) (focal) and negative for cytokeratin 7 (CK7) and estrogen receptor (ER) (Figure 3, A-F). Additionally, progesterone receptor (PR), paired box gene 8 (Pax8), and vimentin were negative (not shown in the figures).

The patient had no history of intestinal malignancy or DES exposure. Colonoscopy performed in 2018 was negative for polyps and no history is suggestive of performing an earlier than the routine screen colonoscopy follow-up. A positron emission tomography (PET) CT scan performed didn't reveal any hypermetabolic lesions other than the primary tumor to suggest metastasis. Tumor markers results were not available. Considering the clinical, radiological, and pathologic findings, a diagnosis of vaginal invasive moderately differentiated adenocarcinoma of intestinal-type was made. The case was also reviewed by Mayo Clinic for consultation. Additional immunostains performed at Mayo Clinic were positive for special AT-rich sequence-binding protein 2 (SATB2), thyroid transcription factor 1 (TTF-1) (patchy), and negative for p16 and GATA3. In situ hybridization for high-risk-human papillomavirus (HPV) E6/E7 is negative. Mayo Clinic is in complete agreement with the original diagnosis. Mismatch repair (MMR) proteins were also tested by immunohistochemical staining on the biopsies in our institution at the clinician's request as a part of working up an adenocarcinoma in the genital tract. The results show no loss of nuclear expression of MMR proteins indicating a low probability of microsatellite instability-high (MSI-H) (not shown in figures).

The patient was counseled about the treatment plan by the interdisciplinary team involved in her management. She started chemoradiation 900 cGy X5 fractions and completed with 4500 cGy X 25 fractions with weekly cisplatin. After that, she underwent interstitial brachytherapy and completed 4.5 Gy X 5 fractions with a Martinez Universal Perineal Interstitial Template (MUPIT) applicator. The patient tolerated the treatment plan very well and denied any side effects. Post-treatment MRI of the pelvis shows a positive response to therapy with minimal if any residual visible tumor. There were minimal post-treatment inflammatory changes. No metastatic disease was identified. Regular follow-up with gynecology oncology and radiation oncology shows no concerning findings. The patient continues with some vaginal discharge, no active discomfort and some vaginal bleeding appreciated with the use of her vaginal dilator. She reported regular bowel movements and no difficulty with urination. She plans to follow up again after six months.

## Discussion

Primary vaginal carcinomas only account for 1% to 2% of all gynecological malignancies with squamous cell carcinoma being the most common histological subtype accounting for 80% to 85% of primary vaginal malignancies [1,2]. Adenocarcinomas account for 15% of vaginal carcinomas and the other 5% of vaginal carcinomas are melanomas, lymphomas, or sarcomas [2]. Subtypes of vaginal adenocarcinomas include clear cell (most common), endometroid, serous, and mucinous [3]. Clear cell variant can occur in the setting of background vaginal adenosis or in women with a history of in-utero exposure to diethylstilbestrol (DES) [5]. Mucinous adenocarcinomas can be further subdivided into endocervical and intestinal subtypes [3]. The intestinal subtype has been reported to arise from vaginal tubular or villous adenomas, adenosis, cloacal remnants, foci of endometriosis, mesonephric duct rests, dysplastic enteric epithelium secondary to surgical manipulation or intestinal metaplasia which was proposed to be due to chronic injury or obstruction in elderly women [1,6-8].

In addition, diagnosis of primary vaginal adenocarcinoma requires the exclusion of metastases from other sites [9,10]. Data show that most of these metastases are from adjacent organs including cervix, endometrium, or ovary, or distant sites including the colon, breast, and pancreas [1,9]. The use of immunohistochemical stains would be helpful in such cases; adenocarcinoma from the endometrium and ovary would be positive for Pax8 but was negative in our case [11]. Cervical adenocarcinoma would be positive for p16 and CEA and negative for ER and vimentin [12,13]. In our case, the patient had a history of normal pap smears without any abnormalities on both physical exam and imaging making cervical adenocarcinoma highly unlikely. Differentiating primary vaginal adenocarcinoma of intestinal-type from metastasis from the gastrointestinal tract is very challenging as they have similar morphologic features as well as immunohistochemical profiles [3]. In our case, the involvement of the periurethral area suggested a possible origin from Skene glands which can undergo intestinal metaplasia [3]. However, no background intestinal metaplasia was identified on the biopsy specimen due to the limited amount of tissue available. Similarly, no area of tubular or villous adenomas or adenosis was present. Careful examination of surgical resection specimens may reveal the origin of histogenesis of this intestinal-type vaginal adenocarcinoma.

Due to the limited diagnostic power of IHC in differentiating primary from metastatic adenocarcinoma of the vagina, clinical and radiological correlation is critical in this scenario [2]. In our case, the patient had no history of an intestinal malignancy, and no occult malignancy was identified on imaging. By the MRI, neither the genitourinary organs including the bladder, uterus, fallopian tubes and ovaries nor the rectum was seen involved in a neoplastic process. The pelvic examination under anesthesia (EUA) and cystoscopy performed during the biopsy of the vaginal mass revealed no bladder lesions and normal cervix. A pelvic MRI and an abdominal scan showed no significant abnormality. A PET CT scan didn't reveal any metastatic disease. In 2019, a screening mammogram showed benign breasts. Colonoscopy performed in 2018 was negative for polyps. These investigations revealed no other primary malignancies which further supported that the vagina might be the primary site of this tumor in this case. Other investigations may be performed as appropriate such as gastroscopy, proctosigmoidoscopy, and serum CEA level.

Data from the literature (Table 1) showed that most of the patients were diagnosed between the third and sixth decade with variable lesion sizes and different sites within the vagina. The immunohistochemical staining pattern mostly resembles that of a gastrointestinal tumor which would make this diagnosis challenging yet provide a clue toward it in the appropriate clinical and radiological contexts. The DES exposure was not present in most of the reported cases we reviewed although some of them did not report the status or had an unknown status of exposure.

**Table 1:** A chronologically-ordered literature review of reported cases of primary vaginal adenocarcinoma of intestinal-type CEA: Carcinoembryonic antigen, EMA: Epithelial membrane antigen, CK20: Cytokeratin 20, CK7: Cytokeratin 7, ER: Estrogen receptor, PR: Progesterone receptor, CDX2: Caudal-type homeobox 2, SATB2: Special AT-rich sequence-binding protein 2, TTF-1: Thyroid transcription factor 1, HPV: Human papillomavirus, MMR proteins: Mismatch repair proteins

Authors	Patient Age (years)	Tumor Size (cm)	Tumor Site	Immunohistochemical & Other Stains	DES exposure
Fukushima et al. [14] (1986)	32 years	3 x 3 x 2 cm	Distal third of the vaginal wall	Neuroendocrine markers were expressed due to mixed components	Unknown
Fox et al. [6] (1988)	35 years	5 x 2 cm	Left lateral and anterior vaginal wall	Unknown	Unknown
Yaghsezian et al. [15] (1992)	52 years	1 cm	Posterior distal vaginal wall	Unknown	Not present
Nagar et al. [16] (1999)	36 years	Depth only: 0.1 cm	Upper anterior vaginal wall	Not reported	Not present
Heller et al. case 1 [17] (2000)	55 years	7 x 4 cm	Anterior lower vaginal wall	CEA+, EMA+	Not present
Heller et al. case 2 [17] (2000)	52 years	2.5 cm	Anterior lower vaginal wall	CEA+, EMA+	Not present
Mudhar et al. [18] (2001)	56 years	1 cm	Distal posterior vaginal wall	CEA+, CK20+, CK7-	Not present
Tjalma and Colpaert et al. [19] (2006)	55 years	4.5 x 4 x 2.7 cm	Anterior lower and posterior vaginal walls	CEA+, CK20+, CK7+, ER-, PR-	Unknown
Ditto et al. [1] (2007)	53 years	1 x 2 cm	Distal posterior vaginal wall	CK20+, CDX2+, CK7-	Not present
Driss et al. [20] (2007)	70 years	4 cm	Anterior lower and middle vaginal walls	CEA+, CK20+, EMA+, CK7-	Not present
van Wessel et al. [21] (2013)	68 years	1 x 1.3 cm	Posterior wall of vaginal introitus	CEA+, CK20+, CK7+	Not present
Staats et al. [22] 10 cases (2014)	Range: 36 to 86 years	Range: 0.8 to 1.5 cm	Variable	CEA+, CK20+, CDX2+, CK (+/-), ER-, PR-	Not present in most cases, few could not be excluded
Tatsumi et al. [3] (2015)	64 years	2 cm	Posterior vaginal wall	CK20+, CDX2+, CK7+ (focal), ER-	Present
Broggi et al. [23] (2018)	51 years	2 cm	Posterior wall of vaginal introitus	CEA+, CK20+, CDX2+, CK7+ (focal), ER-, PR-	Not reported
Ugwu et al. [9] (2019)	40 years	6 x 3 cm	Posterior wall of the lower-third of the vagina with extension to the introitus	Not reported	Not reported
Current case (2022)	62 years	Bilobed components: 1.8 cm, and 2.3 cm	Bilobed components: Middle one-third of the vagina, and lower one-third of the vagina (respective to size)	CEA+, CK20+, CDX2+, EMA+ (focal), SATB2+, TTF-1+ (patchy) CK7-, ER-, PR-, p16-, GATA3- In-situ hybridization for high-risk HPV – MMR proteins -	Not present

The prognosis depends on the patient’s age, tumor stage, tumor differentiation, lymph node status, and distant metastasis [22]. Several treatment modalities are present depending on the tumor stage [22].

## Conclusions

Primary vaginal adenocarcinoma of intestinal-type remains a diagnostic challenge for pathologists since there is no way to rule out primary gastrointestinal malignancy origin with secondary vaginal metastasis based only on pathologic findings. We report another case of this challenging entity to enrich the literature with more cases, in hopes of delivering a better understanding and clearer diagnostic methodology for this neoplasm. It is important to have a high index of clinical suspicion based on presentation, physical findings, and radiologic workup to exclude or diagnose this neoplasm.
